# NLRP3 Inflammasome: A New Target for the Treatment of CVD and Depression Comorbidity

**DOI:** 10.1155/mi/4330574

**Published:** 2025-10-28

**Authors:** Chen Chen, Siqi Zhang, Ming Sheng, Wei Shao

**Affiliations:** ^1^First Clinical Medical College, Hubei University of Chinese Medicine, Wuhan, Hubei, China; ^2^Department of Neurology, Wuhan No. 1 Hospital/Wuhan Hospital of Traditional Chinese and Western Medicine, Wuhan, Hubei, China

**Keywords:** CVD, depression, NLRP3 inflammasome

## Abstract

Depression and cardiovascular disease (CVD) frequently coexist, significantly impacting patient prognosis and quality of life. Research indicates that inflammatory responses play a crucial role in the pathogenesis of both conditions. The NLRP3 inflammasome, a key inflammatory signaling platform of the innate immune system, mediates the maturation and release of IL-1β and IL-18 and induces pyroptosis, playing a significant role in both depression and CVD. To explore the mechanisms and therapeutic potential of the NLRP3 inflammasome in the comorbidity of depression and CVD, we systematically reviewed recent literature. Our focus was on its activation pathways, expression changes in animal models and clinical samples, and intervention studies. The results indicate that NLRP3 inflammasome is persistently activated in patients with both depression and CVD, and this activation correlates with disease severity. Furthermore, various pharmaceutical drugs and natural compounds have demonstrated synergistic effects by inhibiting the NLRP3 inflammasome pathway. In conclusion, the NLRP3 inflammasome represents a critical molecular mechanism linking depression and CVD, as well as a potential target for combined therapeutic strategies. This area holds significant research and clinical translational value.

## 1. Introduction

Depression is a common mood disorder with a continuously rising global prevalence, affecting approximately 350 million people [1]. The World Health Organization (WHO) estimates that around 280 million people worldwide suffer from depression [2], which has become a leading cause of disability and imposes a huge economic burden [3].

Cardiovascular disease (CVD), a collective term for conditions like coronary heart disease and heart failure (HF) [4], poses a major global health threat [5]. In 2019, CVD caused 17.9 million deaths, accounting for 32% of global mortality [6]. Numerous studies have shown a physiological link between depression and CVD [7, 8]. The prevalence of depression is significantly higher in CVD patients, while depression also increases the risk of CVD and is a key risk factor in the progression of the disease [9, 10].

Although there is clear evidence of the comorbidity between CVD and depression, the underlying mechanisms for their pathogenesis are still largely unknown. Among various hypotheses proposed, the inflammation hypothesis has been extensively studied, showing that inflammation plays a crucial role in the pathophysiology of both depression and CVD [11]. The inflammasome is central to this inflammatory response [12]. The NLRP3 inflammasome is the most widely studied in noninfectious diseases [13], and its abnormal activation is closely related to the pathogenesis of both CVD and depression [14]. Therefore, regulating and intervening in the activation of the NLRP3 inflammasome offers new avenues for treating CVD and depression comorbidity.

This review summarizes the role of the NLRP3 inflammasome in the mechanisms of CVD-depression comorbidity and explores the potential and challenges of targeting the NLRP3 inflammasome for the treatment of this condition.

## 2. The Bidirectional Relationship Between Depression and CVD

Epidemiological studies demonstrate that patients with depression have a significantly higher risk of CVD, which can be up to three times that of the general population [15]. A number of studies have confirmed that the prevalence of CVD is markedly increased in patients with major depressive disorder (MDD), and this trend exists across different age groups [16]. For example, a US cohort study showed that the incidence of CVD in MDD patients was nearly three times higher than in healthy controls [17–19]. Furthermore, a meta-analysis showed that the adjusted risk of developing CVD in patients with depression increased by 60% [20]. Long-term follow-up studies have found a positive correlation between the severity of depression and the risk of CVD, suggesting that depression can be considered an independent risk factor for CVD [21]. This is particularly true during the recovery period after an acute event, such as HF with comorbid depression, where the risk of death or rehospitalization within 3–12 months of discharge is 2–3 times higher [22–24].

Conversely, the prevalence of depression is significantly higher in CVD patients than in the general population. Early studies found that nearly 40% of patients with acute myocardial infarction (AMI) experienced depressive symptoms [25], with the incidence varying based on disease severity [26]. For instance, the rate of depression in HF patients increases with their New York Heart Association (NYHA) functional class, rising from 11% in Class I to 42% in Class IV [27]. The potential mechanisms by which CVD leads to depression include psychological stress, reduced quality of life, and biological factors like elevated inflammatory cytokines [28–30]. Studies have shown that even MDD patients with no history of CVD have elevated inflammatory biomarkers, suggesting that depression itself can promote the progression of CVD [31].

The cardiovascular effects of antidepressants remain controversial. Some selective serotonin reuptake inhibitors (SSRIs) show cardioprotective effects, such as anti-inflammatory properties, inhibition of platelet aggregation, and improved endothelial function [32, 33]. However, other studies suggest that certain antidepressants may increase the risk of atrial fibrillation (AF) [34] and further exacerbate cardiovascular burden at high doses. In addition, some antidepressants can affect lipid metabolism and induce myocarditis, thereby increasing CVD risk [35]. Anti-inflammatory therapies may offer a new strategy for managing CVD-depression comorbidity. For example, the IL-1β monoclonal antibody canakinumab has been shown to significantly reduce the risk of recurrent CVD events [36]. Therefore, it is crucial to carefully select antidepressants for CVD patients and monitor their long-term cardiovascular effects.

The high comorbidity between depression and CVD, coupled with a significantly increased mortality rate in co-morbid patients [37], underscores the importance of this relationship. As the severity of depression increases, so do the risks of CVD and associated mortality [16, 26]. Further research into the mechanisms of this interaction will help to optimize clinical intervention strategies.

## 3. The Role of Inflammation in the Comorbidity of CVD and Depression

**Figure 1:**
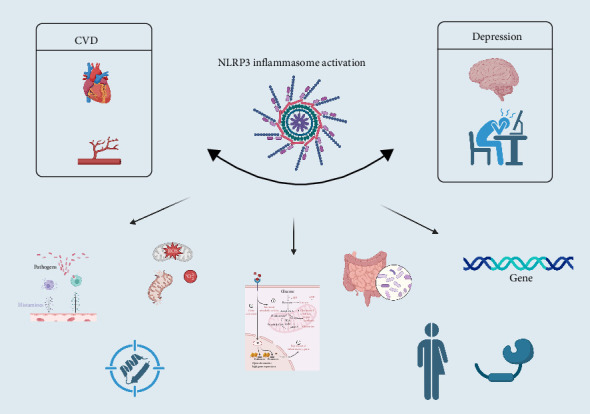
A central therapeutic target in depression-CVD comorbidity.

The mechanisms underlying the comorbidity of depression and CVD are complex, with the inflammatory pathway being extensively studied and considered crucial to the pathophysiology of both conditions [11]. Epidemiological evidence shows a strong link between depression and inflammation; for example, inflammatory biomarkers are significantly elevated in the peripheral blood of some patients with depression [38–40]. Similarly, the prevalence of MDD is markedly higher in patients with chronic inflammatory diseases like inflammatory bowel disease, CVD, and stroke [41]. Furthermore, the inflammatory mechanisms of CVD are gaining increasing attention. Atherosclerosis, for instance, is not just a result of lipid deposition, but also involves a significant inflammatory component [42]. Inflammatory hematological indicators from a complete blood count (CBC) have even been identified as potential prognostic factors for CVD mortality [43].Therefore, regulating and intervening in the activation of the NLRP3 inflammasome offers a new perspective for treating CVD-depression comorbidity (Figure 1).

### 3.1. Overview of the NLRP3 Inflammasome

The inflammasome is an intracellular multiprotein complex integral to the immune response against both pathogen infections and sterile damage. Among the various types of inflammasomes, the NLRP3 inflammasome has been the most extensively studied, with its abnormal activation closely associated with the pathogenesis of CVD and depression [14]. The NLRP3 inflammasome is primarily composed of three components: the NLRP3 receptor protein, the ASC adaptor protein, and the effector enzyme caspase-1. It is predominantly expressed in immune cells, including monocytes, macrophages, and dendritic cells [44]. Activation of the NLRP3 inflammasome promotes the maturation and release of interleukins (IL)-1β and IL-18, subsequently triggering an inflammatory response [45]. Additionally, caspase-1 cleaves gasdermin D (GSDMD), leading to a form of programed cell death known as pyroptosis [46, 47].

### 3.2. Activation Mechanisms of the NLRP3 Inflammasome

**Figure 2:**
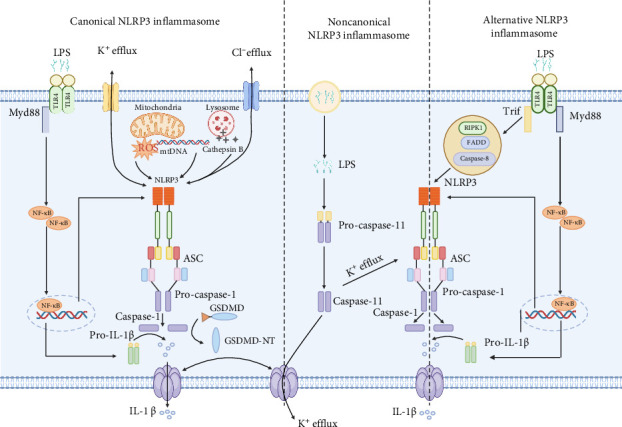
NLRP3 inflammatory vesicle activation pathway.

The activation of the NLRP3 inflammasome primarily occurs through three distinct signaling pathways: the canonical, noncanonical, and alternative pathways. The canonical pathway involves a two-step process comprising a priming phase and an activation phase (Figure 2).

During the priming phase, toll-like receptors (TLRs) or NOD-like receptors (NLRs) initiate a signaling cascade via MyD88 and the NF-κB pathway, resulting in the upregulation of NLRP3 and its precursor proinflammatory cytokines, pro-IL-1β and pro-IL-18 [48, 49]. Notably, the E3 ubiquitin ligase TRIM31 mitigates excessive activation by promoting K48-linked ubiquitination and degradation of NLRP3 [50].

The activation phase is triggered by pathogen-associated molecular patterns (PAMPs) or danger-associated molecular patterns (DAMPs), inducing the assembly of the active NLRP3 inflammasome complex. Key signals for this activation include K^+^ efflux, reactive oxygen species (ROS) generation, and mitochondrial dysfunction, which can be prompted by extracellular ATP and pore-forming toxins [51–53]. This process leads to the oligomerization of the NLRP3 protein, which subsequently assembles with ASC and procaspase-1 to form the inflammasome, ultimately facilitating the maturation and secretion of IL-1β and IL-18 [54, 55].

#### 3.2.1. Mitochondrial Dysfunction as a Core Mechanism of NLRP3 Activation

Mitochondrial dysfunction, a type of DAMP signal, is critical in activating the canonical NLRP3 inflammasome pathway [56]. This effect is particularly pronounced under pathological conditions, such as myocardial ischemia-reperfusion injury and chronic stress [57].

Damaged mitochondria serve as a direct source of signals for NLRP3 inflammasome activation. When the structural integrity of mitochondria is compromised, their membrane permeability increases, leading to the release of internal molecules such as mitochondrial DNA (mtDNA) and cardiolipin into the cytoplasm, which directly activates NLRP3 inflammasome. mtDNA, as an endogenous DAMP, can trigger the assembly and activation of the inflammasome by binding to TLR9 or directly interacting with NLRP3 [58]. Moreover, cardiolipin has been shown to bind directly to NLRP3, causing a conformational change that promotes its aggregation and activation [59, 60]. This mechanism establishes a direct link between mitochondrial damage and the downstream inflammatory response, representing a common driving force for CVD and neuroinflammation.

Various CVD and chronic stress stimuli can lead to mitochondrial dysfunction and subsequent impaired autophagy, resulting in the accumulation of damaged mitochondria in the cytoplasm [61, 62]. The persistent release of molecules from these mitochondria continually stimulates NLRP3 inflammasome, creating a vicious cycle that exacerbates the inflammatory response.

There exists a bidirectional relationship between mitochondrial dysfunction and the NLRP3 inflammasome. On one hand, excessive activation of the NLRP3 inflammasome can contribute to mitochondrial dysfunction. For instance, NLRP3 gene deletion has been shown to alleviate mitochondrial abnormalities, myocardial cell apoptosis, and cardiac fibrosis [63]. Conversely, in microglia of the central nervous system (CNS), mitochondrial dysfunction and impaired mitophagy can lead to persistent NLRP3 inflammasome activation, forming a harmful feedback loop of oxidative stress and inflammation [64].

#### 3.2.2. Other Activation Pathways

The noncanonical pathway is induced by Gram-negative bacteria. Their LPS can directly activate human caspase-4/5 or mouse caspase-11, which cleaves GSDMD and induces pyroptosis. Once activated, caspase-11 promotes pannexin-1-mediated ATP release and K^+^ efflux, which in turn stimulates IL-1β and IL-18 secretion through the NLRP3-ASC-caspase-1 pathway [65, 66].

The alternative pathway can be activated by a single TLR ligand signal. For example, the TLR4-TRIF-RIPK1-FADD-CASP8 axis can directly activate the NLRP3 inflammasome to release IL-1β [67, 68]. However, this pathway does not induce K^+^ efflux, ASC speck formation, or pyroptosis.

### 3.3. Common Mechanisms of NLRP3 Inflammasome in the Comorbidity of CVD and Depression

The NLRP3 inflammasome functions as a critical link between CVD and depression, with shared underlying mechanisms in several areas.

#### 3.3.1. Core Role of the Inflammatory Pathway

As an essential component of the innate immune system, the NLRP3 inflammasome exhibits excessive activation in both CVD and depression. This activation results in the release of proinflammatory cytokines, such as IL-1β and IL-18, leading to a state of chronic low-grade inflammation that serves as a fundamental pathological basis for the comorbidity of these diseases [14, 69, 70]. Studies have demonstrated that elevated levels of NLRP3 in patients with MI are significantly associated with an increased risk of depression [71].

#### 3.3.2. Oxidative Stress and Pathway Crossover

ROS are critical molecules that connect NLRP3 activation to both diseases. ROS can activate the NLRP3 inflammasome via the p38 MAPK signaling pathway, a mechanism validated in cases of arsenic-induced depression and ischemic heart disease [72, 73]. Simultaneously, NLRP3 inflammasome inhibitors can mitigate oxidative stress and inflammation, thus establishing a bidirectional regulatory loop [72].

#### 3.3.3. Trained Immunity and Sustained Activation

Trained immunity refers to the long-term functional reprograming of innate immune cells following a transient stimulus, resulting in a more robust response to subsequent stimuli [74]. This “memory” effect may account for the sustained low-level activation of NLRP3 inflammasome observed in both depression and CVD. Initial stimuli, such as chronic stress, infections, or metabolic disorders, can induce epigenetic modifications in macrophages, leading to enhanced transcription of inflammatory genes, including NLRP3 and IL-1β [75]. Even after the initial stimulus subsides, this epigenetic memory can drive an excessive and persistent inflammatory response to minor subsequent triggers [76]. Thus, trained immunity, by keeping the NLRP3 inflammasome in a highly activatable state, may constitute a vital mechanism linking chronic inflammation with the pathogenesis of both conditions.

#### 3.3.4. Regulatory Role of the Gut Microbiome

The gut microbiome is considered a crucial axis connecting depression and CVD. Dysbiosis in gut microbiota can activate the host's NLRP3 inflammasome through various pathways, influencing both cardiovascular and neurological health [12, 77]. For instance, a dysbiotic gut flora may produce bacterial components such as LPS, which can infiltrate the bloodstream and activate NLRP3 inflammasome, leading to systemic inflammation [78]. Additionally, a decrease in beneficial microbial metabolites, such as short-chain fatty acids, or an increase in harmful metabolites, is linked to NLRP3 inflammasome activation. Notably, TMAO has been shown to activate the microglial NLRP3 inflammasome via the FTO/IGF2BP2 pathway, exacerbating neurological damage following ischemic stroke [79]. Moreover, gut microbiota dysbiosis can impair nervous system function by stimulating the NLRP3 inflammasome and releasing inflammatory cytokines, thereby regulating the “gut-brain axis” and exacerbating depressive symptoms [80].

#### 3.3.5. Shared Genetic and Molecular Basis

Genetic analyses have revealed that MDD and atherosclerotic CVD share numerous genetic risk factors, with several risk loci implicating NLRP3-related pathways [81]. Activation of the NLRP3/IL-1β pathway may concurrently worsen cardiovascular damage and neuropsychiatric symptoms [82, 83].

#### 3.3.6. Sex- and Age-Related Inflammatory Susceptibility

The prevalence of both CVD and depression exhibits significant variations based on sex and age, which are closely correlated with the regulation of the NLRP3 inflammasome. Estrogen is recognized as a critical regulator of NLRP3 activation. Research indicates that estrogen inhibits NLRP3 inflammasome activation through various mechanisms, offering women a degree of inflammatory protection during their reproductive years [84]. Nonetheless, the decline in estrogen levels postmenopause increases women's susceptibility to NLRP3-mediated inflammation [85], consequently raising the incidence of both CVD and depression [86]. Additionally, aging is known to be a proinflammatory factor. Over time, chronic inflammation accumulates, partly due to the persistent upregulation of NLRP3 activity [85]. Inflammatory factors secreted by senescent cells, along with mitochondrial dysfunction and impaired autophagy, contribute to sustained NLRP3 inflammasome activation, thereby accelerating the development and progression of cardiovascular and depressive conditions [87].

#### 3.3.7. Overlapping Therapeutic Targets

Substances such as natural polyphenols, N-acetylcysteine, and MCC950 show therapeutic promise for both CVD and depression by inhibiting NLRP3 inflammasome activation [73, 88, 89]. Specifically, the triterpenoid compound 1,2,4-TTB can concurrently suppress hippocampal NLRP3 inflammasome activation and improve depressive-like behaviors [89]. Collectively, these mechanisms establish an “inflammation-oxidative stress-neuroendocrine” network, elucidating why the risk of depression escalates sharply with the complexity of cardiovascular complications [90]. Therefore, interventions targeting NLRP3 inflammasome may offer a novel strategy to disrupt this vicious cycle [69, 91].

## 4. The Role of NLRP3 in Depression

### 4.1. The Relationship Between NLRP3 and Depression

The neuroinflammatory hypothesis of depression posits that neuroinflammatory pathways play a crucial role in the onset and progression of the disorder [92, 93]. The NLRP3 inflammasome is regarded as a principal mediator in the development of depression, given its widespread expression in neurons, microglia, and astrocytes [94, 95]. The inflammation and immune responses mediated by the NLRP3 inflammasome are significant contributors to psychiatric disorders [96], potentially leading to behavioral changes associated with depression and cognitive impairment [97].

### 4.2. Changes in the NLRP3 Inflammasome in Patients With Depression

#### 4.2.1. Clinical Evidence

A substantial body of research indicates that excessive peripheral inflammation can compromise the integrity of the blood-brain barrier (BBB), subsequently triggering neuroinflammation in the brain [98]. Moreover, activated NLRP3 inflammasomes have been identified in both brain tissue and peripheral blood samples from patients with depression, along with an overproduction of inflammatory proteins and proinflammatory cytokines (Table 1). The elevation of these inflammatory biomarkers is closely associated with the severity of mood fluctuations and psychiatric symptoms [114, 115].

Furthermore, studies have established a positive correlation between inflammation levels and fluctuations in depressive symptoms. As inflammation intensifies, symptoms tend to worsen, while a decrease in inflammation correlates with symptom improvement [116]. Thus, the activation status of the NLRP3 inflammasome can be assessed by measuring the levels of NLRP3 protein, IL-1β, IL-18, and ASC speck formation in a patient's blood or brain tissue. This assessment may aid in evaluating the severity and prognosis of depression [98, 117]. Additionally, the abnormal activation of the NLRP3 inflammasome not only affects mood, but may also precipitate other complications related to depression [118]. These findings lend support to the hypothesis that depression may arise from an aberrant interaction between the CNS and the innate immune system.

#### 4.2.2. Preclinical Evidence

##### 4.2.2.1. Stress-Induced Models

Depression is closely associated with chronic stress, and models of stress-induced depression effectively replicate its etiology, neurobiological changes, and behavioral phenotypes. Among these models, the chronic social defeat stress (CSDS) model exhibits significant construct validity on a social level, closely resembling human depressive pathology [119]. Research indicates that CSDS can activate the NLRP3 inflammasome, resulting in increased levels of NLRP3, IL-1β, and caspase-1 in brain tissue [120].

Similarly, the chronic unpredictable mild stress (CUMS) model has been shown to induce depressive-like behaviors in mice while activating the NLRP3 inflammasome in both the hippocampus and serum, thereby elevating IL-1β expression. Notably, the deletion of the NLRP3 gene can abolish these effects [121, 122]. Furthermore, chronic restraint stress (CRS), social defeat, and early-life stress are all capable of triggering NLRP3-mediated hippocampal inflammation, leading to the upregulation of NLRP3, IL-1β, and IL-18 expression, potentially inducing pyroptosis in neurons or microglia [123–125].

##### 4.2.2.2. Pharmacological Models

4.2.2.2.1. *LPS Model* LPS model is widely utilized to examine inflammation-related depression [126]. Intraperitoneal injection of LPS induces neuroinflammation, resulting in depressive-like behaviors and cognitive alterations [127, 128]. LPS activates the NF-κB pathway via TLR4, which promotes the release of proinflammatory cytokines and triggers hippocampal NLRP3 inflammasome activation through the TRPV4-CaMKIIα signaling pathway [129]. Following LPS administration, the expression levels of NLRP3, ASC, and caspase-1 increase [130–132]. Inflammasome inhibitors can reverse the elevation of IL-1β and IL-18 levels, thereby alleviating depressive-like behaviors [133], indicating that the NLRP3 inflammasome serves as a key mediator.

*4.2.2.2.2. Reserpine (RESP) Model* The RESP-induced depression model is frequently employed to investigate the effects of monoamine depletion on depression [134–136]. Although a direct association between RESP and the NLRP3 inflammasome has not been established, research indicates that RESP administration upregulates the expression of NLRP3, caspase-1, and IL-1β in the hippocampus of mice [137]

*4.2.2.2.3. Corticosterone (CORT) Model* Excessive activation of the hypothalamic-pituitary-adrenal (HPA) axis, resulting in elevated CORT secretion, is recognized as a crucial mechanism underlying depression [53, 107]. Prolonged exposure to high levels of CORT can induce depressive-like behaviors and may activate the NLRP3 inflammasome through HPA axis dysfunction [138]. Proinflammatory factors stimulate the HPA axis, leading to cortisol release, which diminishes the peripheral immune system's sensitivity to anti-inflammatory feedback, creating a vicious cycle [100]. Research has demonstrated that repeated CORT administration can increase the expression of NLRP3, ASC, caspase-1, IL-18, and IL-1β in the prefrontal cortex and hippocampus [116, 139], resulting in an immune cascade, neuroplasticity deficits, and neuronal damage [140]. Furthermore, CORT-induced NLRP3 activation can lead to pyroptosis in PC12 and HT22 cells [139, 141].

##### 4.2.2.3. Surgical Models

*4.2.2.3.1. Poststroke Depression (PSD) Model* The mechanisms underlying PSD are complex, with inflammation playing a significant role in its development [142, 143]. Studies have shown that inflammatory cytokine levels are elevated in both PSD patients and animal models, accompanied by the activation of the TLR4/p38/NF-κB/NLRP3 pathway [144, 145]. NLRP3 activation and pyroptosis are significantly elevated in the brain tissue of PSD models [146, 147]. In a Mongolian gerbil PSD model, the expression of NLRP3, cleaved caspase-1, mature IL-1β, IL-18, GSDMD-N, and ASC were all upregulated [148]

*4.2.2.3.2. High-Fat Diet (HFD)-Induced Model* A HFD can activate hippocampal microglia, induce inflammation, and impair hippocampal function, thereby increasing the risk of cognitive impairment [149, 150]. Increased NLRP3 activation and IL-1β expression have been detected in the hippocampus and amygdala of HFD-fed mice, which may contribute significantly to cognitive impairment [150, 151]. In HFD-induced depression models, the expression of NLRP3, ASC, caspase-1, and IL-1β is markedly elevated [152]. Peripheral NLRP3 agonists can exacerbate cognitive deficits in HFD-fed mice, promote hippocampal IL-1β and IL-1R expression, and induce microglial damage and apoptosis [151]

Across various animal models of depression, the NLRP3 inflammasome has been demonstrated to play a crucial role in the disease's pathophysiology. Stressors, pharmacological agents, and surgical interventions can all activate NLRP3, resulting in neuroinflammation, pyroptosis, and neuronal damage, which provides important evidence for the inflammatory mechanisms of depression(Table 2).

### 4.3. The Molecular Basis by Which NLRP3 May Lead to Depression

The activation of the NLRP3 inflammasome is intricately linked to the pathophysiology of depression [159]. In the absence of NLRP3, chronic stress fails to activate microglia in mice or elicit depressive-like behaviors [160]. Research indicates that upon the activation of NLRP3, factors such as GSDMD, IL-1β, and IL-18 collectively contribute to the pathogenesis of depression.

GSDMD is a protein that promotes pyroptosis. Upon NLRP3 activation, caspase-1 cleaves GSDMD, and the resulting GSDMD-N terminal fragment forms pores in the cell membrane. This process leads to the release of proinflammatory factors, instigating inflammation and pyroptosis [161, 162]. In a CUMS mouse model, the NLRP3/caspase-1/GSDMD pathway was shown to mediate pyroptosis in hippocampal astrocytes [163]. Notably, deletion of the GSDMD gene can reduce pyroptosis and ameliorate depressive-like behaviors [164].

IL-1β is a pivotal activator of the HPA axis [165, 166]. Its proinflammatory effects can result in cellular damage, with heightened IL-1β signaling associated with resistance to antidepressant treatments [167, 168]. Chronic stress activates the NLRP3 inflammasome, leading to IL-1β release, which induces systemic inflammation and precipitates depressive-like behaviors [138]. Elevated IL-1β levels are observed in patients with MDD [169, 170]. The NLRP3 inflammasome is primarily expressed in microglia and serves as a critical driver of neuroinflammation [85, 171]. Following stress, activated microglia release inflammatory factors that instigate neuroinflammation and facilitate the emergence of anxiety and depression [172]. By activating microglia, IL-1β disrupts neurotrophic functions and induces depressive-like behaviors [173]. Furthermore, deletion of the IL-1β gene can alleviate LPS-induced depressive-like behaviors [174].

IL-18, an immune-regulatory factor, stimulates Th1 cells to produce IFN-γ and is positively correlated with NLRP3 mRNA expression [175]. As a member of the IL-1 superfamily, IL-18 can stimulate the synthesis of TNF-α and IL-1β [176] and has been linked to schizophrenia [177]. IL-18 contributes to the development of depression by regulating the HPA axis [178] and is recognized as a biomarker of psychological stress, with elevated levels detected in the blood of individuals with depression [179, 180]. High IL-18 levels may heighten susceptibility to poststress depression [181] and interact with serotonin (5-HT) to affect brain function and trigger emotional disorders [182]. In mouse models, abnormal IL-18 levels are associated with depressive-like behaviors, and alterations in hippocampal structure correlate with the intensity of depressive symptoms [183, 184]. While IL-18-deficient mice exhibit resistance to stress, they also demonstrate obesity, dyslipidemia, nonalcoholic fatty liver disease, and depressive-like behaviors [185, 186]. Recombinant IL-18 may promote neurogenesis [183], yet administering exogenous IL-18 into the amygdala can precipitate severe depressive-like behaviors [187]. The exact mechanism underlying IL-18's influence on depressive-like behavior requires further exploration.

### 4.4. Targeting NLRP3 for Depression Treatment: The Application of NLRP3 Inhibitors

The NLRP3 inflammasome is critically implicated in the pathophysiology of depression. Modulating the NLRP3/ASC/caspase-1/GSDMD/IL-1β/IL-18 axis presents significant clinical potential for developing new antidepressant therapies [102]. Currently, various strategies to treat depression by targeting the NLRP3 inflammasome have emerged, including NLRP3 pathway inhibitors, natural compounds, other therapeutic agents, and nonpharmacological approaches.

#### 4.4.1. Traditional Antidepressant Medications

Multiple classes of traditional antidepressants, such as SSRIs, tetracyclic antidepressants (TeCAs), serotonin-norepinephrine reuptake inhibitors (SNRIs), and tricyclic antidepressants (TCAs), inhibit the activation of the NLRP3 inflammasome in MDD (Table 3). For instance, fluoxetine, an SSRI, has been found to inhibit NLRP3 inflammasome activation by downregulating the ROS-PKR-NLRP3 signaling pathway, which in turn reduces IL-1β secretion and ameliorates depressive-like behaviors induced by chronic mild stress [189]. Additionally, fluoxetine exerts neuroprotective effects by modulating CNS inflammation via the TLR4/NF-κB/NLRP3 signaling pathway [194]. Furthermore, SSRIs can alleviate astrocyte loss associated with pyroptosis through the NLRP3/caspase-1/GSDMD pathway [164].

#### 4.4.2. Ketamine

In addition to traditional antidepressants, ketamine—a noncompetitive NMDA receptor antagonist—demonstrates anti-inflammatory properties and elicits rapid antidepressant effects, particularly in patients with treatment-resistant depression [195, 196]. Ketamine exerts these effects by modulating the NLRP3 inflammasome pathway and increasing AMPA receptor levels [197].

Empirical studies have shown that ketamine can alleviate depressive symptoms by countering LPS-induced overexpression of both NLRP3 and IL-1β [198]. In a CRS model, ketamine induced microglial autophagy, inhibiting NLRP3 inflammasome activation, reducing IL-1β levels, and reversing depressive-like behaviors [199]. Nevertheless, the therapeutic window for ketamine is narrow; very low doses (0.1 mL/kg) lack efficacy, whereas excessively high doses (20–100 mg/kg) may activate the NLRP3/caspase-1 axis, potentially leading to neuronal pyroptosis [200–202].

#### 4.4.3. NLRP3 Inflammasome Inhibitors and Their Antidepressant Effects

NLRP3 inflammasome inhibitors effectively target and suppress NLRP3 activity, thereby reducing the levels of inflammatory factors (Table 4). CY-09, a small-molecule NLRP3 inflammasome inhibitor, blocks inflammasome activation by inhibiting its ATPase activity, thus exhibiting antidepressant effects [203]. MCC950 inhibits the activation of both canonical and noncanonical NLRP3 inflammasomes by blocking the ATPase domain of NLRP3. This action reduces the protein levels of NLRP3, IL-1β, and IL-18 in the hippocampus, consequently preventing depressive symptoms in mice induced by chronic social isolation [204]. Additionally, MCC950 further inhibits NLRP3 activation by suppressing ATP hydrolysis [209].

Beta-hydroxybutyrate (BHB), a ketone body, inhibits K^+^ efflux, which in turn reduces ASC oligomerization and the release of IL-1β and IL-18 [210]. Cli095 exerts its antidepressant effects by inhibiting the TLR4-NF-κB-NLRP3 pathway [206]. BAY 117,082 inhibits NF-κB, thereby lowering the levels of NLRP3, IL-18, and IL-1β, among other inflammatory factors [138]. P2X7 receptor inhibitors, such as Brilliant Blue G and A438079, alleviate depressive-like behaviors by inhibiting the ATP-P2X7 receptor-NLRP3 signaling pathway [207]. VX-765, a specific caspase-1 inhibitor, prevents the activation of the NLRP3 inflammasome [211]. Similarly, Ac-YVAD-CMK significantly reduces LPS-induced depressive-like behaviors [133, 198].

#### 4.4.4. Natural Compounds

Natural compounds have garnered considerable attention as potential alternatives or adjuncts to antidepressants due to their reduced side effects and favorable tolerability. Numerous natural compounds have exhibited antidepressant effects by inhibiting the activation of the NLRP3 inflammasome in animal studies (Table 5); however, their clinical application necessitates further investigation.

#### 4.4.5. Other Therapeutic Approaches

The NLRP3 inflammasome may also play a role in the onset of depression through the gut-brain axis. Chronic mild stress can induce an imbalance in the gut microbiome, and fecal microbiota transplantation has been shown to alleviate depressive symptoms by inhibiting NLRP3 inflammasome activation [77].

Mesenchymal stem cells (MSCs) possess anti-inflammatory and immunomodulatory properties that can suppress NLRP3 inflammasome activation and inflammatory cytokine secretion, thus improving depressive-like behaviors [220, 221]. Peptides, as biological mediators with notable efficacy and low toxicity, may offer a novel therapeutic option for depression. For instance, the Moschus polypeptide (PPM) mitigates LPS-induced inflammation by inhibiting the NF-κB-ROS/NLRP3 pathway [222].

Nonpharmacological treatments targeting the NLRP3 inflammasome, such as physical exercise, acupuncture, and electroacupuncture, have also demonstrated effectiveness in treating depression. Physical exercise can enhance depressive-like behaviors by reducing NLRP3 inflammasome activation in ovariectomized mice [223] and can inhibit the TLR4/NF-κB/NLRP3 signaling pathway in the hippocampus of mice in a PSD model [224]. Acupuncture exerts its antidepressant effects by regulating the NLRP3 inflammasome in the prefrontal cortex of rats with chronic stress-induced depression [225]. Similarly, electroacupuncture can prevent chronic mild stress-induced depressive-like behaviors by modulating the NLRP3 inflammasome in the hippocampus [226].

## 5. The Role of the NLRP3 Inflammasome in CVD

### 5.1. The Relationship Between NLRP3 and CVD

The NLRP3 inflammasome is instrumental in the pathogenesis of CVD, particularly in conditions such as atherosclerosis, ischemia/reperfusion (I/R) injury, and HF [227]. Both conventional and novel therapeutic agents have been shown to exert their effects via the NLRP3 inflammasome. Key components of this pathway, including NLRP3, caspase-1, and IL-1β, are considered promising targets for CVD treatment [228].

### 5.2. NLRP3 Dynamics in CVD

#### 5.2.1. Clinical Evidence

Research indicates that activation of the NLRP3 inflammasome is critical in CVD, particularly concerning atherosclerosis and MI(Table 6). In atherosclerotic plaques, mRNA expression levels of NLRP3 and its associated genes (e.g., *ASC*, *CASP1*, *IL1B*, *and IL18*) show significant increases and correlate with the severity of plaque lesions [229]. In patients with hypertension, diabetes, and tobacco use, aortic NLRP3 expression is elevated and positively correlates with total cholesterol and low-density lipoprotein cholesterol, while negatively correlating with high-density lipoprotein cholesterol [230]. Additionally, NLRP3 mRNA levels are higher in plaques from symptomatic patients compared to those who are asymptomatic, suggesting that NLRP3 may serve as a biomarker for disease progression [229]. Furthermore, aortic NLRP3 expression is significantly elevated in patients undergoing coronary artery bypass grafting (CABG) compared to healthy controls, closely correlating with the severity of coronary atherosclerosis [230].

In patients with MI, concentrations of NLRP3 inflammasome and associated inflammatory factors are markedly elevated in peripheral blood samples, particularly following myocardial ischemia-reperfusion injury, indicating that NLRP3 may serve as a prognostic indicator [231]. The activity of the NLRP3 inflammasome is also associated with cell types implicated in pulmonary hypertension and systemic hypertension [234]. Interventions such as aerobic exercise can enhance cardiovascular metabolic status and related disease outcomes by inhibiting NLRP3 inflammasome activation and decreasing IL-1β levels [235]. This evidence underscores the significance of NLRP3 inflammasome activation as a pivotal factor in the progression and prognosis of CVD, highlighting its potential as a critical target for future therapeutic interventions.

#### 5.2.2. Preclinical Evidence

##### 5.2.2.1. Atherosclerosis

Atherosclerosis is a chronic inflammatory disease characterized by the infiltration of immune cells, lipid accumulation, and the proliferation of vascular smooth muscle cells, ultimately resulting in the formation of atherosclerotic plaques. Research indicates that components of the NLRP3 inflammasome are significantly elevated in carotid atherosclerotic plaques, highlighting the critical role of NLRP3 in the pathogenesis of atherosclerosis [236]. The activation of NLRP3 in endothelial cells (ECs) represents a key event leading to endothelial damage [227], and its impact on atherosclerosis is primarily mediated by the effector cytokine IL-1β [237, 238]. Elevated expression levels of NLRP3 are closely associated with the severity of coronary artery stenosis [229]. Additionally, inflammasome-mediated cholesterol efflux is important in the progression of atherosclerosis [227].

In murine models, the expression of inflammasome components such as NLRP3, ASC, caspase-1, IL-18, and IL-1β is markedly increased in the aorta [239]. Knockout of NLRP3, ASC, and IL-1α/β significantly mitigated early atherosclerosis and IL-18 levels when subjected to a Western-style high-cholesterol diet [240]. Moreover, mice deficient in NLRP3, ASC, or caspase-1/11 exhibited reduced atherosclerotic lesions following transplantation [227, 240]. The absence of IL-1β also significantly alleviates atherosclerosis in apoE-deficient mice [237].

##### 5.2.2.2. HF

The NLRP3 inflammasome is intricately linked to the onset and progression of HF, the final stage of many cardiac dysfunctions and a leading cause of CVD-related mortality. In patients experiencing acute decompensated systolic HF, contractility is compromised through both IL-1- and IL-18-dependent pathways [241]. Regulation of the NLRP3 inflammasome can mitigate cardiac hypertrophy, enhance diastolic and systolic function, and inhibit the progression of HF. In pressure overload models that replicate the pathological alterations associated with HF, NLRP3 inflammasome expression is heightened in transverse aortic constriction (TAC) mouse models. Conversely, myocardial cell-specific CaMKIIδ deletion can diminish NLRP3 inflammasome activity, subsequently relieving ventricular dilation and systolic dysfunction, while also reducing cardiac fibrosis and remodeling [242].

Some studies, however, propose that *NLRP3* deficiency may compromise cardiac function by increasing the expression of TLR4, potentially resulting in heightened inflammation, fibrosis, and hypertrophy in pressure overload models. This suggests a possible negative regulatory role for NLRP3 in cardiac remodeling [243]. Consequently, the precise mechanisms underlying NLRP3's involvement in HF remain to be fully elucidated and warrant further exploration.

##### 5.2.2.3. Arrhythmia

The NLRP3 inflammasome is pivotal in the development of arrhythmias, particularly in the context of HF and AF. HF patients frequently undergo electrical remodeling, which can precipitate arrhythmias [244]. Studies have demonstrated that activation of TLR2 and the NLRP3 inflammasome in cardiac macrophages can instigate IL-1β production in diabetic mice [245]. IL-1β promotes oxidative stress and protein kinase C (PKC) activation, which decreases the density of L-type Ca_2_^+^ channels, leading to arrhythmias [246]. Persistent inflammation can also lead to myocardial fibrosis, exacerbating the condition [247]. In a CREM-TG mouse model, NLRP3 inflammasome activity was significantly elevated, indicating a strong association with the incidence of AF [248].

##### 5.2.2.4. Hypertension

Hypertension is a significant risk factor for CVD, with inflammation playing a critical role in its pathogenesis. Studies have indicated that the NLRP3 inflammasome in animal models of hypertension can promote atherosclerosis and myocardial injury by facilitating immune cell infiltration, triggering oxidative stress, and inducing vascular endothelial dysfunction [249, 250]. In hypertensive mice, activation of the NLRP3 inflammasome leads to the proliferation of vascular smooth muscle cells and vascular remodeling [251], along with the promotion of proinflammatory cytokine secretion [252]. Additionally, noncoding RNAs, such as miR-1929-3 p, can inhibit NLRP3 activation in macrophages, thereby improving cardiac remodeling associated with hypertension [253]. These findings suggest that the NLRP3 inflammasome plays a multifaceted regulatory role in the development of hypertension, positioning it as a potential therapeutic target.

##### 5.2.2.5. AMI

AMI is an acute ischemic heart disease caused by inadequate coronary blood flow, with inflammation being a primary mechanism underlying myocardial injury [254]. Research has identified the NLRP3 inflammasome as crucial in the development and adverse remodeling following myocardial infarction. Inflammatory mediators, including IL-1β and IL-18, contribute to the pathogenesis of myocardial infarction [255]. In murine models of AMI, activation of NLRP3, ASC, and caspase-1 occurs, resulting in the release of IL-1β and IL-18, while activation in myocardial cells leads to pyroptosis [256]. Silencing NLRP3 or the P2X7 receptor has been shown to reduce infarct size following an AMI [257]. Furthermore, in a myocardial I/R injury model, mice lacking NLRP3 exhibited a significant reduction in infarct size, an effect not mirrored in mice lacking ASC [258]. These results suggest that inhibition of the NLRP3 inflammasome could represent a viable therapeutic strategy for alleviating the inflammatory response and preserving myocardial function following myocardial infarction (Table 7).

### 5.3. The Molecular Basis by Which the NLRP3 Inflammasome May Contribute to CVD

Activation of the NLRP3 inflammasome is intricately linked to the pathogenesis of CVD. Research indicates that in mice deficient in NLRP3, the levels of pro-inflammatory cytokines IL-1β and IL-18 are significantly reduced, resulting in markedly smaller atherosclerotic lesions [267]. The activation of the NLRP3 inflammasome can accelerate the progression of CVD by promoting foam cell formation, inducing macrophage pyroptosis, and enhancing vascular inflammation. The primary mechanisms underlying these processes involve the IL-1β, IL-18, and GSDMD-dependent pyroptosis pathways, as elaborated below.

In NLRP3 inflammasome-mediated CVD, the activation of GSDMD is closely associated with the progression and rupture of atherosclerotic plaques [268]. GSDMD binds to cardiolipin on the mitochondrial membrane, thus activating the cGAS-STING signaling pathway, which promotes the secretion of inflammatory mediators such as IL-1β, further exacerbating inflammation and accelerating plaque formation [269]. For example, in a mouse model of atherosclerosis induced by a HFD, the protein levels of NLRP3, ASC, GSDMD-N, IL-1β, and IL-18 were significantly elevated; GSDMD-mediated pyroptosis then accelerated the progression of atherosclerosis [270]. Moreover, GSDMD-deficient mice exhibited smaller atherosclerotic lesions [271]. In an AMI model, GSDMD activation facilitated IL-1β release and worsened myocardial inflammation. Specific knockout of GSDMD significantly reduced infarct size while improving cardiac function and survival rates [272].

IL-1β is a pivotal innate immune factor that primarily interacts with ECs, smooth muscle cells, and macrophages, enhancing the expression of leukocyte adhesion molecules and thrombogenic factors [273]. In the context of CVD, IL-1β drives disease progression by activating the inflammatory response, aggravating myocardial injury, and promoting scar tissue formation [274]. Elevated levels of IL-1β are closely associated with the progression of atherosclerosis and cardiac fibrosis [275]. The CANTOS trial demonstrated that an IL-1β inhibitor significantly decreased the recurrence rate of cardiovascular events, further substantiating IL-1β's central role in CVD [276]. Additionally, inhibition of IL-1β can mitigate cardiac fibrosis and improve heart function [277].

IL-18 is a critical proinflammatory cytokine within the NLRP3 signaling pathway that exacerbates CVD progression by activating multiple inflammatory pathways [278]. IL-18 enhances the expression of adhesion molecules such as E-selectin, ICAM-1, and VCAM-1, and augments the secretion of inflammatory mediators like IL-6 and CRP, thereby worsening atherosclerosis and myocardial injury [279]. An ApoE-/- mouse model demonstrated that exogenous IL-18 exacerbated atherosclerotic lesions, while inhibition of IL-18 resulted in reduced plaque area and promoted the formation of stable plaques [280]. Furthermore, an IL-18 neutralizing antibody exhibited significant therapeutic effects in an AMI model, reducing infarct size and improving cardiac function [281]. Caspase-1 serves as a downstream effector enzyme of the NLRP3 inflammasome, facilitating the activation of IL-1β and IL-18. Elevated levels of IL-1β and IL-18 in atherosclerotic plaques are closely linked to inflammasome activation [282]. Studies have shown that deficiencies in caspase-1 can significantly diminish atherosclerotic lesions in ApoE-/- mice [283] and reduce plaque area [267]. Thus, caspase-1 inhibitors may represent a novel therapeutic strategy for CVD by lowering the levels of proinflammatory factors.

### 5.4. Targeting NLRP3 for CVD Treatment: The Application of NLRP3 Inhibitors

Statins, metformin, dapagliflozin, colchicine, NLRP3 inhibitors, and various natural compounds have been shown to inhibit the activation of the NLRP3 inflammasome in CVD. These medications suppress NLRP3 inflammasome activation and the secretion of proinflammatory factors through distinct mechanisms [284–290].

#### 5.4.1. Conventional Cardiovascular Medications and Risk Factors

Statins exhibit anti-inflammatory effects by reducing the expression of NLRP3 and its downstream factors. For instance, atorvastatin significantly decreases the production of NLRP3, IL-1β, and caspase-1, contributing to the stabilization of atherosclerotic plaques and the reduction of lesion size [291–293]. Similarly, simvastatin lowers IL-1β expression, thereby decreasing cardiovascular risk [285].

Metformin inhibits NLRP3 inflammasome activation by enhancing the activity of AMP-activated protein kinase (AMPK) and protein phosphatase 2A (PP2A), which in turn reduces NLRP3 expression. Furthermore, it promotes autophagy by inhibiting the AMPK/mTOR pathway, resulting in improved outcomes in diabetic cardiomyopathy and the preservation of cardiac function [286, 294].

Dapagliflozin improves diabetic cardiomyopathy by inhibiting NLRP3 inflammasome activation, pyroptosis, and apoptosis while reducing pro-inflammatory factors in a TAC-induced HF model [295, 296].

Both colchicine and dapagliflozin can decelerate the progression of atherosclerosis through the inhibition of NLRP3 inflammasome activation [297, 298].

Melatonin decreases the expression of proinflammatory factors such as IL-1β and caspase-1 by stimulating mitophagy and inhibiting NLRP3 inflammasome activation via the Sirt3/FOXO3 a/Parkin signaling pathway [299].

#### 5.4.2. NLRP3 Inflammasome Inhibitors

Colchicine inhibits the NLRP3 inflammasome through four mechanisms: suppressing gene expression, interfering with tubulin, blocking caspase-1 activity, and reducing potassium (K^+^) efflux. It mitigates the inflammatory response in the infarcted area, improves survival rates, and slows the progression of HF [288, 300]. Clinical studies indicate that colchicine significantly lowers the risk of cardiovascular events in patients with AMI and stable coronary artery disease [290, 301].

MCC950 specifically inhibits the activation of the NLRP3 inflammasome and reduces IL-1β secretion without affecting other inflammasomes, thereby demonstrating its potential to maintain immune balance [302, 303]. This compound may prevent CVD such as hypertension, atherosclerosis, and myocardial infarction [249].

Bay 11-7082 selectively inhibits NLRP3 inflammasome activation by suppressing the NF-κB pathway, which correlates with reduced NLRP3 expression [304].

CY-09 inhibits NLRP3 inflammasome activation by binding to the NACHT domain of NLRP3, thus restricting its oligomerization and the assembly of the inflammasome. This inhibitor is specific to NLRP3 and does not impact the activation of other inflammasomes (Table 8) [318, 319].

#### 5.4.3. Natural Compounds

Natural compounds exhibit promising potential in inhibiting NLRP3 inflammasome activation and treating CVD. Animal studies have shown that specific natural compounds can effectively suppress NLRP3 inflammasome activation, thereby offering new therapeutic avenues for CVD (Table 9).

#### 5.4.4. Other Therapies

Exercise contributes to the improvement of CVD by suppressing NLRP3 inflammasome activation. Research indicates that exercise mitigates inflammation in ECs, prevents the formation of atherosclerotic plaques, and slows the progression of atherosclerosis [331, 332]. In mouse models, aerobic exercise has been found to significantly inhibit the activation of the NLRP3/caspase-1/IL-1β signaling pathway, resulting in reduced myocardial hypertrophy and fibrosis [333]. Furthermore, exercise positively impacts the mitigation of myocardial injury following ischemia-reperfusion [257].

In summary, through the modulation of NLRP3 inflammasome activity, various traditional drugs and targeted inhibitors can effectively improve CVD and its associated complications. Future research may further substantiate the clinical applicability of these strategies.

## 6. The NLRP3 Inflammasome: A New Therapeutic Target for Comorbid Depression and CVD and its Clinical Significance

The NLRP3 inflammasome, a critical immune signaling platform, has been extensively investigated as a promising therapeutic target for the comorbidity of depression and CVD. Evidence indicates that the NLRP3 inflammasome significantly contributes to the pathogenesis of depression by activating proinflammatory cytokines such as IL-1β and IL-18, which also play pivotal roles in the development of CVD. Through interactions with inflammatory pathways, the NLRP3 inflammasome not only facilitates the progression of depression but may also aggravate the pathological processes associated with CVD, thereby creating a vicious cycle that intensifies disease symptoms. These findings suggest that targeting the NLRP3 inflammasome could provide valuable therapeutic strategies, particularly for the prevention and early intervention of both conditions [334, 335].

As a therapeutic target, the activation of the NLRP3 inflammasome is central to the shared pathology of depression and CVD. Thus, inhibiting the NLRP3 inflammasome may be critical in alleviating the worsening comorbidity of these two diseases. Previous studies have demonstrated that NLRP3 inhibitors can significantly ameliorate pathological manifestations in models of depression and CVD, underscoring their potential as dual therapeutic targets. These inhibitors function by preventing NLRP3 inflammasome activation, which reduces the release of associated inflammatory factors and may effectively mitigate disease symptoms.

Moreover, inflammatory factors linked to the NLRP3 inflammasome could serve as potential biomarkers for the early diagnosis and monitoring of treatment for depression-CVD comorbidity. These biomarkers not only facilitate the differential diagnosis of the conditions but also provide a robust basis for assessing treatment efficacy.

With respect to intervention strategies, research indicates that NLRP3 inflammasome inhibitors can significantly reduce depressive-like behaviors in animal models and lower the risk of CVD. This presents an opportunity to develop novel therapeutic approaches, especially given that conventional antidepressants and CVD treatments often exhibit limited effectiveness. By integrating NLRP3 inflammasome inhibitors with existing treatments, it may be feasible to enhance the management of both CVD and depressive symptoms while concurrently reducing the overall inflammatory burden, thereby improving overall therapeutic outcomes.

In conclusion, the NLRP3 inflammasome represents not only a central molecular hub linking the comorbidity of depression and CVD, but also a promising therapeutic target that merits further research and clinical investigation. Nonetheless, despite initial evidence of its therapeutic potential, challenges to clinical translation remain, as discussed below.

## 7. Obstacles and Strategies for Clinical Translation of NLRP3 Therapeutics, and Limitations of Current Preclinical Models

### 7.1. Obstacles to Clinical Translation

#### 7.1.1. Off-Target Effects and Immunosuppression Risk

The therapeutic promise of NLRP3 inhibitors in preclinical studies highlights their potential for anti-inflammatory and tissue-protective effects. However, these inhibitors may inadvertently disrupt the compensatory activation of other inflammasomes, potentially exacerbating disease progression in unanticipated ways [336]. For instance, some inhibitors may interact with conserved domains of NLRP3 and simultaneously impact other inflammatory pathways, complicating therapeutic outcomes [337].

#### 7.1.2. Lack of Patient Stratification Strategies

A significant barrier to the successful clinical application of NLRP3 inhibitors is the absence of reliable biomarkers and clinical stratification techniques capable of accurately pinpointing patient cohorts most likely to benefit from these therapies. This lack of stratification leads to considerable variability in clinical trial outcomes, as diverse patient populations with differing disease etiologies may respond in unique ways to NLRP3 inhibition [336]. Current models inadequately capture this complexity, limiting predictive power [337–339].

#### 7.1.3. Limitations of Early-Stage Clinical Validation

Although preliminary investigations indicate a favorable safety profile for NLRP3 inhibitors, the absence of ongoing monitoring and the scale limitations of initial studies hinder a comprehensive understanding of long-term safety and potential adverse effects [340].

### 7.2. Limitations of Preclinical Models

#### 7.2.1. Species Differences and Inadequate Pathological Replication

The significant discrepancies between the immune systems and disease processes of animal models and humans pose a challenge for translating preclinical findings into clinical contexts [341]. For instance, chemically induced Alzheimer's disease (AD) models lack the intricate pathological features characteristic of the human condition, resulting in limited applicability of therapeutic insights [342].

#### 7.2.2. Complexity of Inflammasome Activation Mechanisms

Preclinical studies often simplify NLRP3 inflammasome activation by employing a singular stimulus; however, in human diseases, NLRP3 activation is typically multifaceted, involving various pathways that preclinical models struggle to accurately replicate.

#### 7.2.3. Inability to Simulate Dynamic Disease Progression

Existing preclinical models primarily focus on acute injury mechanisms, yet diseases associated with the NLRP3 inflammasome often exhibit chronic characteristics. As a result, these models may fail to reflect the long-term pathological changes associated with sustained NLRP3 activation.

### 7.3. Future Strategies

#### 7.3.1. Optimizing Drug Delivery Systems

Advancements in nanocarrier technologies could enhance the specificity of NLRP3 inhibitor delivery, thereby mitigating off-target effects. Research into modulating surface chemical properties for the purpose of nonspecific NLRP3 activation inhibition illuminates new avenues for targeted drug delivery system design [343]. Additionally, employing local scaffold diversity generators may significantly enhance drug bioavailability [344]. Given the potential for compensatory inflammasome activation, developing joint inhibitors that cotarget NLRP3 alongside other pathways, or combining them with existing antidepressants or cardiovascular medications, could prove beneficial.

#### 7.3.2. Multi-Omics and Biomarker Development

The integration of multi-omics data models has already shown remarkable accuracy in the staging and classification of various diseases [345]. We propose enhancing these models by combining single-cell sequencing with proteomics to identify specific biomarkers indicative of NLRP3 activation, which could inform patient stratification.

#### 7.3.3. Developing Humanized Models and Organoid Technology

The utilization of human cell-derived organoids or transgenic animal models, integrated with clinical samples, holds promise for improving the pathological relevance of preclinical studies. Innovations like the MOBER technology can augment clinical predictability through transcriptome correction, advancing our understanding of NLRP3-related diseases [346].

#### 7.3.4. Simulating Disease Microenvironments and Assessing Broad-Spectrum Inhibitors

By mimicking the complex microenvironments of human diseases through the combined application of diverse activators, we can gain insight into the efficacy of molecules currently undergoing clinical trials within multipathway models. This approach may facilitate the discovery of broad-spectrum inhibitory capabilities, thereby enriching the validity of preclinical simulations in reflecting the complexities inherent in human diseases.

#### 7.3.5. Validating With Long-Term Intervention Models

To accurately represent chronic pathological states in humans, the establishment of long-term intervention models is essential. These models may involve sustained stimulation or the continuous low-dose administration of proinflammatory factors, allowing for a more nuanced understanding of disease progression and treatment efficacy over extended periods.

## Figures and Tables

**Table 1:** Changes in NLRP3 inflammasome in patients with depression.

Patients	NLRP3		ASC		Caspase-1		IL-1β		IL-18		References
	Changes	Region	Changes	Region	Changes	Region	Changes	Region	Changes	Region	
MDD patients	Increase	Serum	—	—	Increase	Serum	—	—	—	—	[99]
MDD patients	Increase	Serum	—	—	—	—	Increase	Serum	Increase	Serum	[100]
MDD patients	—	—	—	—	—	—	Increase	CSF	—	—	[101]
MDD patients	Increase	Serum	—	—	—	—	Increase	Serum	Increase	Serum	[102]
MDD patients	—	—	—	—	—	—	Increase	Serum	—	—	[29, 101, 103–107]
PPD patients	Increase	Serum	—	—	Increase	Serum	—	—	—	—	[101]
Suicide patients with depression	Increase	mPFC	Increase	mPFC	Increase	mPFC	Increase	mPFC	Increase	mPFC	[108]
BD patients	—	—	—	—	—	—	Increase	Prefrontal cortex	—	—	[109]
BD patients	—	—	—	—	—	—	Increase	CSF	—	—	[110]
BD patients	Increase	PBMC	Increase	PBMC	Increase	PBMC	Increase	PBMC	Increase	PBMC	[111]
Depression patients		—	Increase	PBMC	—	—	—	—	—	—	[112]
Depression patients	Increase	PBMC	—	—	—	—	—	—	—	—	[113]

**Table 2:** Alternation of NLRP3 inflammatory vesicles in animal models of depression.

Model	NLRP3		ASC		Caspase-1		IL-1β		IL-18		References
	Changes	Region	Changes	Region	Changes	Region	Changes	Region	Changes	Region	
CRS	Increase	Hippocampus	Increase	Hippocampus	—	—	Increase	Serum	—	—	[153]
CRS	Increase	Hippocampus	—	—	Increase	Hippocampus	Increase	Hippocampus	Increase	Hippocampus	[154]
CRS	Increase	—	—	—	Increase	—	Increase	—	—	—	[155]
CUMS	Increase	Hippocampus	Increase	Hippocampus	Increase	Hippocampus	Increase	Hippocampus	Increase	Hippocampus	[156]
CORT	Increase	Hippocampus	Increase	Hippocampus	Increase	Hippocampus	Increase	Hippocampus	Increase	Hippocampus	[139]
RESP	Increase	Hippocampus	—	—	Increase	Hippocampus	Increase	Hippocampus	—	—	[116]
PSD	Increase	Hippocampus	Increase	Hippocampus	Increase	Hippocampus	Increase	Hippocampus	Increase	Hippocampus	[137]
HFD	Increase	Hippocampus	Increase	Hippocampus	Increase	Hippocampus	Increase	Hippocampus	—	—	[148]
CSDS	Increase	Hippocampus	Increase	Hippocampus	Increase	Hippocampus	Increase	Hippocampus	—	—	[152]
LPS	Increase	mPFC	—	—	—	—	Increase	mPFC	—	—	[157]
LPS	Increase	Hippocampus	Increase	Hippocampus	Increase	Hippocampus	Increase	Hippocampus	—	—	[130]
LPS	Increase	Hippocampus	Increase	Hippocampus	Increase	Hippocampus	Increase	Hippocampus	Increase	Hippocampus	[131]
LPS	Increase	—	—	—	Increase	—	Increase	—	Increase	—	[132]
LPS	Increase	Hippocampus	—	—	Increase	Hippocampus	Increase	Hippocampus	—	—	[154, 158]

**Table 3:** Pharmacological activity of traditional antidepressants in treating depression by targeting the NLRP3 inflammasome.

	Antidepressant	Samples	Effects	Reference
SSRIs	Fluoxetine	mPFC of rats exposed to CUMS	Reduction of IL-1β levels and inhibition of NLRP3 inflammatory vesicles and NF-κB pathway	[188]
		Hippocampus of C57 BL/6 mice exposed to CMS	Fluoxetine inhibits NLRP3 inflammatory vesicle activation by controlling ROS production and NLRP3 binding to PKR	[189]
		Peripheral blood in patients with MDD	Levels of proinflammatory cytokines IL-1β and NLRP3 were significantly reduced	[190]
		PBMC from patients with MDD	Inhibition of NLRP3-inflammatory vesicle activation by enhanced autophagy	[102]
		Serum from mice exposed to RS	Reduced IL-1β serum levels	[102]
	Paroxetine	BMC from patients with MDD	Inhibition of NLRP3-inflammatory vesicle activation by enhanced autophagy	[102]
		Serum from mice exposed to RS	Reduced IL-1β serum levels	[102]
	Citalopram	Hippocampus of mice exposed to CMS stimulation	Reduction of NLRP3/caspase-1/GSDMD pathway-dependent astrocyte focal death	[164]

TCAs	Amitriptyline	BMC from a patient with MDD	Inhibition of NLRP3 inflammatory vesicle activation through enhanced autophagy	[102]
		Serum from mice exposed to RS	Reduced IL-1β serum levels	[102]
		Serum from MDD patients	IL-18 and IL-1β were significantly reduced, and NLRP3 and caspase-1 activation were significantly reduced	[179]
	Clomipramine	Hippocampus of CMS-exposed mice	Inhibition of the microglia marker Iba-1, modulation of NLRP3 inflammatory vesicles and proinflammatory factors to increase hippocampal volume	[191]
		Serum from LPS-induced depression mouse model	Decreased ASC, the active component of NLRP3 inflammatory vesicles, inhibited the proinflammatory process induced by LPS administration, resulting in improved depression-like behavior	[192]

TeCA	Mianserin	BMC from MDD patients	Inhibits NLRP3-inflammatory vesicle activation by enhancing autophagy	[102]
		Serum from mice exposed to RS	Reduces IL-1β serum levels	[102]

NaSSA	Mirtazapine	BMC from MDD patients	Inhibits NLRP3-inflammatory vesicle activation by enhancing autophagy	[102]
		Serum from RS-exposed mice	Reduced IL-1β serum levels	[102]

SNRI/	Venlafaxine	Prenatal stress-exposed rats	Reduced IL-1β levels inhibit NLRP3-inflammatory vesicle activation	[193]
		BMC from MDD patients	Inhibits NLRP3-inflammatory vesicle activation by enhancing autophagy	[102]
		RS-exposed mouse serum	Reduced IL-1β serum levels	[102]

Melatonin	Agomelatine	BMC from MDD patients	Inhibits NLRP3-inflammatory vesicle activation by enhancing autophagy	[102]
		Serum from RS-exposed mice	Reduces IL-1β serum levels	[102]

NMDA	Esketamine	Hippocampal tissue from LPS-induced depression model mice	Low-dose aketamine ameliorates LPS-induced depressive symptoms in mice by modulating the GSK-3β/NLRP3 pathway	[158]

**Table 4:** Pharmacological activity of drugs targeting the NLRP3 inflammatory vesicle pathway as potential treatments for depression.

Type	Name	Samples	Effects	Reference
NLRP3 inflammatory vesicle inhibitor	CY-09	LPS-induced hippocampus of male ICR mice	CY-09 inhibits NLRP3/ASC/cytokine signaling pathway and microglia activation	[203]
	MCC950	Chronic social isolation-induced hippocampus of male C57BL/6J mice	MCC950 significantly reduced the levels of NLRP3, IL-1β, and IL-18	[204]
	BHB	CUMS-induced prefrontal cortex of male Sprague-Dawley rats	BHB downregulates the levels of proinflammatory cytokines	[205]

TLR4 inhibitor	CLI 095	CUMS-induced prefrontal cortex and hippocampus of male ICR mice	Cli-095 inhibits TLR4-NF-κB-NLRP3 signaling pathway	[206]

NF-κB inhibitor	BAY 117,082	Dexamethasone-induced HAPI cell model	BAY 117,082 reduces the levels of NF-κB, IL-1β, IL-18, NLRP3, ASC, and cleaved caspase-1	[138]

P2X7 receptor inhibitor	Brilliant Blue G	CUMS-induced hippocampus of male Sprague-Dawley rats	Brilliant Blue G inhibits the ATP-P2X7 receptor-NLRP3 signaling pathway	[207]
	A438079	UMS-induced hippocampus of male Sprague-Dawley rats	A438079 inhibits the ATP-P2X7 receptor-NLRP3 signaling pathway	[207]

Caspase-1 inhibitor	VX-765	CUMS-induced serum and hippocampus of male BALB/c mice	VX-765 downregulates serum and hippocampal IL-1β levels	[121]
		Ovariectomy-induced hippocampus of female C57BL/6J mice	VX-765 downregulates serum and hippocampal IL-1β levels	[208]

**Table 5:** Pharmacological activity of natural compounds targeting the NLRP3 inflammatory vesicle pathway as a potential treatment for depression.

Name	Samples	Effects	Reference
Astragalus polysaccharide	CRS-induced prefrontal cortex in ICR mice	Inhibits the activation of NLRP3/ASC/caspase-1/IL-1β signaling pathway and reduces the release of proinflammatory cytokines	[212]

Tetramethylpyrazine	Prefrontal cortex and hippocampus of CUMS-induced ICR mice	Inhibits the TLR4-NF-κB-NLRP3 signaling pathway	[206]

Betaine	Lipopolysaccharide-induced hippocampus of ICR mice	Inhibited NLRP3 inflammatory vesicle activation to promote microglia transition from M1 to M2 phenotype	[213]

Andrographolide	CMS-induced prefrontal cortex of C57BL/6 mice	Reduced expression of proinflammatory mediators and cytokines and NLRP3 inflammatory vesicle assembly in prefrontal cortex	[214]

Crocin	LPS-induced BV-2 microglia	Inhibited NLRP3 inflammatory vesicles and NF-κB and their promotion of microglia M1 to M2 phenotypic transition	[215]

Pinosylvin	LPS-induced hippocampus and frontal lobe of C57BL/6J mice	Reduces hippocampal NLRP3 inflammatory vesicle expression and downregulates the netrin-1/DCC signaling pathway	[216]

Gypenoside L	LPS-induced hippocampus and prefrontal cortex of C57BL/6 mice	Inhibits the NLRP3/caspase-1/ASC signaling pathway	[217]

Ginsenoside	CMS-induced hippocampus of C57BL/6J mice	Blocked stress-induced activation of NLRP3 inflammatory vesicles in microglia	[218]

Salvianolic acid B	CMS-induced Wistar rat serum	Inhibits NLRP3 inflammatory vesicle activation to abrogate oxidative stress and neuroinflammation in the hippocampus	[219]

Salidroside	LPS or CORT-induced hippocampus of C57BL/6 mice	Ameliorates focal death and reverses LPS-induced NLRP3 activation by inhibiting the P2X7/NF-κB/NLRP3 pathway	[139]

**Table 6:** Changes in NLRP3 inflammatory vesicles in CVD patients.

Type	NLRP3		ASC		Caspase-1		IL-1β		IL-18		Reference
	Changes	Region	Changes	Region	Changes	Region	Changes	Region	Changes	Region	
Atherosclerosis	Increase	Carotid atheroselerosis	Increase	Carotid atheroselerosis	Increase	Carotid atheroselerosis	Increase	Carotid atheroselerosis	Increase	Carotid atheroselerosis	[229]
CABG	Increase	Aorta	—	—	—	—	—	—	—	—	[230]
MI	Increase	Blood	—	—	—	—	—	—	—	—	[231]
HIV-ASCVD	—	—	—	—	Increase	Blood	—	—	—	—	[232]
ACS	Increase	Plasma	—	—	—	—	—	—	—	—	[233]

**Table 7:** Alternation of NLRP3 inflammatory vesicles in animal models of CVD.

Model	NLRP3		ASC		Caspase-1		IL-1β		IL-18		References
	Changes	Region	Changes	Region	Changes	Region	Changes	Region	Changes	Region	
Atherosclerosis	Increase	Aorta	Increase	Aorta	Increase	Aorta	Increase	Aorta	Increase	Aorta	[239]
Atherosclerosis	Increase	Aorta	—	—	Increase	Aorta	—	—	—	—	[259]
Atherosclerosis	Increase	Aorta	Increase	Aorta	Increase	Aorta	Increase	Aorta	—	—	[260]
TAC	Increase	Myocardium	—	—	—	—	Increase	Myocardium	Increase	Myocardium	[242]
Cardiac insufficiency from ASO	Increase	Myocardium	Increase	Myocardium	Increase	Myocardium	—	—	Increase	Myocardium	[261]
I/R	Increase	Myocardium	—	—	Increase	Myocardium	Increase	Myocardium	Increase	Myocardium	[262]
MI	Increase	Myocardium	Increase	Myocardium	Increase	Myocardium	Increase	Myocardium	Increase	Myocardium	[263]
TAC	Increase	Myocardium	Increase	Myocardium	Increase	Myocardium	—	—	—	—	[264]
CREM-TG (spontaneous AF)	Increase	Cardiac atrium	Increase	Cardiac atrium	Increase	Cardiac atrium	—	—	—	—	[248]
SHR	Increase	Vascular smooth cell	Increase	Vascular smooth cell	Increase	Vascular smooth cell	Increase	Vascular smooth cell	—	—	[265]
MI	Increase	Myocardium	—	—	Increase	Myocardium	Increase	Myocardium	Increase	Myocardium	[266]

**Table 8:** Pharmacological activity of drugs targeting the NLRP3 inflammatory vesicle pathway as potential treatments for CVD.

Type	Name	Samples	Effects	Reference
NLRP3 inflammatory vesicle inhibitor	Colchicine	Myocardium of mice with acute myocardial infarction	Significantly reduced NLRP3 inflammatory vesicle activation and NLRP3 downstream focal death-related protein expression	[300, 305]
	CY-09	Blood vessels in diabetic mice	Limits NLRP3 oligomerization and inflammatory vesicle assembly	[306]
	MCC950	Salt-induced hypertension	Lowers blood pressure	[307]
		Angiotensin II infusion in Camk2 dfl/fl mice	Reduced macrophage accumulation and cardiac fibrosis	[308]
		High fat, high cholesterol, and angII-treated mice	Inhibits excitation-induced aortic dilatation, entrapment, and rupture in thoracic and abdominal aortic segments	[86]
		Atherosclerosis	Reduced NLRP3, caspase-1 expression, and IL-1β secretion and slowed down the development of atherosclerotic lesions	[302, 309, 310]
		Atherosclerosis	Reduce the expression of adhesion molecules in plaques and the number of macrophages in plaques	[311]
		Myocardial infarction in pigs	Reduced neutrophil infiltration, lowered myocardial IL-1β levels, reduced infarct size, and preserved cardiac function	[312]
		Mice with permanent coronary artery occlusion	Reduced inflammatory cell infiltration, caspase-1 activation, IL-18 and IL-1β levels, and myocardial fibrosis improved cardiac remodeling	[313]

NF-κB inhibito	BAY 117082	Ischemia/reperfusion models	—	[304, 314, 315]
		Myocardial injury after ischemia/reperfusion in diabetic rats	Pretreatment with BAY 117082 reduced inflammatory cell infiltration as well as cardiomyocyte apoptosis and infarct size, and preserved cardiac function	[316, 317]

**Table 9:** Pharmacological activity of natural compounds targeting the NLRP3 inflammatory vesicle pathway as a potential treatment for CVD.

Name	Samples	Effects	Reference
Triptolide	Infusion of LSO-induced cardiac fibrosis mice	Blocking NLRP3 inflammatory vesicle assembly and activation of the NLRP3-TGF-β1-Smad signaling pathway reduced pressure overload-induced cardiac hypertrophy and fibrosis	[320]

Emodin	Myocardial ischemia reperfusion injury rats; LPS-induced septic myocardial injury mice	Inhibition of cardiomyocyte pyroptosis and attenuation of myocardial I/R injury via the TLR4/MyD88/NF-κB/NLRP3 inflammatory vesicle pathway showed anti-inflammatory effects and inhibition of LPS-induced activation of inflammatory vesicles and thus antimyocardial fibrosis	[321, 322]

Artemisinin	HFD-fed ApoE mice	Ability to inhibit the inflammatory response through the AMPK/NF-κB-NLRP3 pathway, thus exhibiting vasoprotective properties	[323]

Curcumin	Azithromycin (DOX)-induced myocardial injury mice	Inhibits NLRP3 inflammatory vesicle activation, decreases expression of NLRP3, caspase-1 and IL-1β, and reduces apoptosis	[324]

Rosmarinic acid	Nicotine-induced atherosclerotic rats	Antiatherosclerotic, inhibits activation of the pyrin structural domain	[325]

Erucic acid	Diabetic atherosclerotic rats	Attenuated NLRP3 inflammatory vesicle activation and pyroptosis	[326]

Gypenoside L	Diabetic cardiomyopathy rats	Inhibited the production of ROS and cytochrome C. Significantly down-regulated the activation of NLRP3 inflammatory vesicles and reduced IL-1β and IL-18 levels	[327]

Huoxue Qianyang Qutan recipe	Spontaneously hypertensive rats (SHRs)	Downregulated the expression of NLRP3, caspase-1, and IL-1β molecules in obese SHR, ameliorated hypertension and inhibited myocardial fibrosis	[328, 329]

LuQi formula	Left anterior descending (LAD) coronary artery ligation-induced MI model	Interfered with ROS generation in myocardial infarction mice, thereby attenuating oxidative stress, reducing TXNIP activity, preventing its binding to NLRP3 inflammasome, inhibiting NLRP3 pathway activation, and delaying ventricular remodeling	[330]

## Data Availability

The data that support the findings of this study are within the article. Further data are available from the corresponding author upon reasonable request.
